# Spinal manipulation frequency and dosage effects on clinical and physiological outcomes: a scoping review

**DOI:** 10.1186/s12998-019-0244-0

**Published:** 2019-05-22

**Authors:** Mégane Pasquier, Catherine Daneau, Andrée-Anne Marchand, Arnaud Lardon, Martin Descarreaux

**Affiliations:** 1 Department of Anatomy, Université du Québec à Trois-Rivières, Trois- Rivières, Québec Canada; 2 Institut Franco-Européen de Chiropraxie, Ivry-sur-Seine, France; 3 Department of Human Kinetics, Université du Québec à Trois-Rivières, Trois- Rivières, Québec Canada; 4 CIAMS, Université Paris-Sud, Université Paris-Saclay, Orsay, France; 5 CIAMS, Université d’Orléans, Orléans, France

**Keywords:** Spinal manipulation, Dosage, Frequency, Clinical response, Scoping review

## Abstract

**Introduction:**

The burden of musculoskeletal disorders increases every year, with low back and neck pain being the most frequently reported conditions for seeking manual therapy treatment. In recent years, manual therapy research has begun exploring the dose-response relationship between spinal manipulation treatment characteristics and both clinical and physiological response to treatment.

**Objective:**

The purpose of this scoping review was to identify and appraise the current state of scientific knowledge regarding the effects of spinal manipulation frequency and dosage on both clinical and physiological responses.

**Methods:**

A scoping review was conducted to identify all available studies pertaining to our research question. Retrieved papers were screened using a 2-phase method, a selective sorting with titles and abstracts. Potentially relevant studies were read, and data was extracted for all included studies. Randomized control trials were assessed using the Cochrane Risk of Bias Tool for quality assessment.

**Results:**

The search yielded 4854 publications from which 32 were included for analysis. Results were sorted by dosage or frequency outcomes, and divided into human or animal studies. Animal studies mainly focused on dosage and evaluated physiological outcomes only. Studies investigating spinal manipulation dosage effects involved both human and animal research, and showed that varying thrust forces, or thrust durations can impact vertebral displacement, muscular response amplitude or muscle spindle activity. Risk of bias analysis indicated only two clinical trials assessing frequency effects presented a low risk of bias. Although trends in improvement were observed and indicated that increasing the number of SM visits in a short period of time (few weeks) decreased pain and improve disability, the differences between the studied treatment frequencies, were often not statistically significant and therefore not clinically meaningful.

**Conclusion:**

The results of this study showed that SM dosage and frequency effects have been mostly studied over the past two decades. Definitions for these two concepts however differ across studies. Overall, the results showed that treatment frequency does not significantly affect clinical outcomes during and following a SM treatment period. Dosage effects clearly influence short-term physiological responses to SM treatment, but relationships between these responses and clinical outcomes remains to be investigated.

## Introduction

Musculoskeletal disorders represent a major public health issue. According to the 2016 Global Burden of Diseases Study, back and neck pain rank among the top five disorders with regard to years lived with disability, and the related expenses increase every year [1]. Disability-Adjusted Life Years (DALYs) associated to low back and neck pain keep rising every decade, with an estimated increase of about 30 million people affected between 1990 and 2016. Moreover, a recent special issue published in the Lancet highlighted the fact that disability related to low back pain is projected to increase in low-income and middle-income countries, where resources and quality healthcare are limited, but also where back and neck have been far less studied [2].

Several evidence-based practice guidelines for back and neck pain management have been published in the last decade [3–6]. They clearly highlight the complex nature of back and neck pain clinical management while providing guidance and potential care pathways for patients-clinicians shared decision-making. Although most of these recent guidelines are based on low to moderate evidence, the vast majority of them suggests that conservative treatments, including manual therapies, are effective options to treat acute, subacute, and chronic spinal disorders.

Manual therapies are used by many professionals around the world. Among these therapies, chiropractic is widely used. And according to Beliveau et al., low back pain (49,7%) is the first complaint that drives patients to chiropractic offices, followed by neck pain (22,5%) and extremity disorders (10%) [1, 7]. Spinal manipulation (SM) is defined as a thrust of high velocity and low amplitude delivered to the spine using a specific contact in order to provide mobility to a joint [8]. It is the most common tool used by chiropractors to treat patients as 79% reported using this treatment modality on a regular basis [7]. Although current evidence suggests that SM can yield positive clinical outcomes such as reducing pain and disability, current knowledge regarding the underlying mechanisms leading to such clinical responses is scarce [3, 4].

From a medial perspective, the effectiveness of a treatment is commonly contingent on the patient’s compliance and persistence, which are characterized by the patient adherence to the treatment prescription. The prescription is first defined by the treatment dosage. Dosage conditions the response or the pattern of the physiological response, for which there is a threshold defining the lower and higher dosages that can be prescribed to have a positive effect and to avoid an adverse event [9]. The prescription is also defined by the dose frequency, which is the number of times a substance is administered within a specific time period or the number of doses administered over a specific time interval. Prescription, however, is not a construct commonly used within the context of SM.

Recent studies have showed that SM physiological and biomechanical effects can be characterized based on SM force-time profiles using biomechanical parameters such as thrust force, preload force, thrust duration and rate of force application [10]. However, the relation between dose, frequency and treatment outcome remains unknown.

Moreover, there is no known standard regarding the number of SM treatments that should be administered in the management of a given condition. In clinical practice, the frequency of treatments depends mostly on the clinician’s personal experience. Chiropractors adapt their treatments according to the patients’ symptoms presentation. In fact, their treatments are modulated based on the individual’s conditions and symptoms as well as their also response to treatments. Despite the number of studies published in the past few years trying to define SM treatments focusing on physiological effects of variable dose on animals or variable frequency in human, there is a lack of evidence regarding how many SM treatments over a given period are required and what dosage should be used.

The purpose of this scoping review is therefore to evaluate the current state of scientific knowledge regarding the effect of SM frequency and dosage on both clinical and physiological responses. Specifically, the primary objective is to identify all clinical and physiological outcomes specific to SM frequency and dosages effects. The secondary objective is to report on the clinical and physiological effects of frequency and dosages. The third objective is to document all adverse events.

## Methods

A scoping review was chosen as the most appropriate methodology to answer such a broad research question and capture the breadth of information on a topic that has been studied through diverse and heterogeneous designs. It identifies gaps in current knowledge in order to inform future research studies. This scoping review was based on the framework from Levac et al. using a 5-step method review [11].

### Step 1: identifying the research question

This scoping review was conducted to answer the following research question: what is the current state of scientific knowledge regarding the effect of SM treatment frequency and dosage on both clinical and physiological responses?

### Step 2: identifying relevant studies

The search strategy was developed in collaboration with a university librarian and conducted using the following databases: MEDLINE, CINAHL (Cumulative Index to Nursing and Allied Health Literature), ICL (Index to Chiropractic Literature), MANTIS (Manual, Alternative and Natural Therapy Index System) and Cochrane Central Register of Controlled Trials. Databases were searched from inception up to September 2017. A combination of the following indexing terms (MESH or non-MESH) relevant to our research theme was used: musculoskeletal manipulation, dose-response or dosage, and frequency. The authors also searched for additional data sources from google scholar, conference abstracts and proceedings, references from unpublished data, and book chapters. An Endnote (version X8.2, Clarivate Analytics©, Boston MA, USA) library was created to import all search results and remove any duplicates.

### Step 3: study selection

#### Inclusion and exclusion criteria

To be included, the studies had to be published in a peer-reviewed journal and written in English, French or Spanish language. We considered for inclusion human-based studies (without any age limits) and studies involving animal models. Studies also had to involve any form of SM (including mobilization) as well as a modulation and a quantification of at least one parameter of treatment frequency or dosage. All included studies had to match the following operational definitions for frequency and dosage:

1. SM dosage was defined as any quantified biomechanical parameters derived from the SM force-time profile such as the preload force, the thrust force, and the duration or rate of force application.
2. SM frequency was defined as the number of SM treatment delivered over a given period of time.

To be included, a study had to include within or between group comparison of different SM dosages or different SM treatment frequencies. The following types of publication were excluded: practice guidelines, unpublished manuscripts, dissertations, government reports, books or book chapters, and conference proceedings.

#### Screening and agreement

**Fig. 1:**
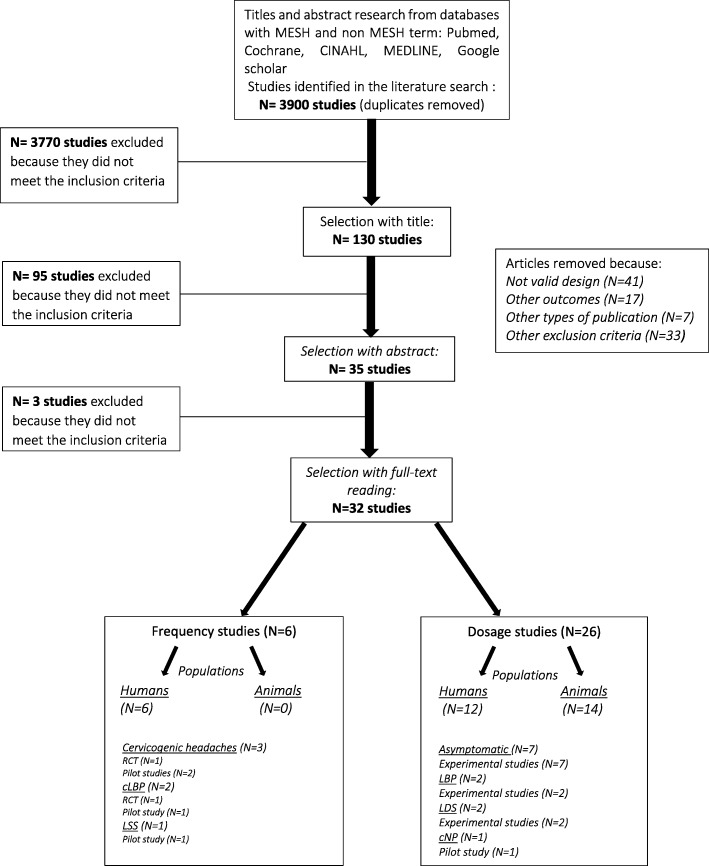
Flowchart diagram. CGH: Cervicogenic Headache; LBP: Low back pain; LSS: Lumbar Spinal Stenosis; LDS: Lumbar Decompression Surgery

We used a 2-phase screening process to select eligible studies. A pair of reviewers (MP, CD) independently screened the search results, using an Excel spreadsheet for both phases. The first phase (I) aimed at determining the study eligibility using titles and abstracts only. Studies were then classified as relevant, possibly relevant and irrelevant. The second phase (II) involved a full-text screening of the relevant and possibly relevant studies to identify the final list of articles from which data was extracted for this review (Fig. 1.). For each of these phases, a third reviewer (AAM) was asked to solve any disagreement during the consensual screening process.

### Step 4: charting the data

In order to extract and sort the data from relevant studies, a Word table was created; it included the following items: *authors and year of publication, study design, purpose of the study, sample size, treatment protocol, groups or experimental conditions, outcomes measures, clinical or physiological effects, and adverse events*. From the papers included in the phase II of the screening process, three articles were selected to test the data extraction table. Data extraction was completed by one investigator (MP) and double-checked by a second investigator (AL), who was not involved in the earlier stages of the selection.

### Step 5: collating, summarizing and reporting the results

A descriptive analysis was made to detail the search results including the number of papers kept for analysis, their year of publication and study design. The summary of the evidence table was divided into two sections, the first half being dedicated to the dosage studies and the second half to the frequency studies. Data for humans and animals were summarized separately due to the nature of the respective study outcomes. In order to provide a quality assessment of randomized control trials (RCT), all frequency studies (six publications) were submitted to the Cochrane Risk of bias tool [12]. Two assessors (MP and AAM) independently evaluated the following items: *random sequence generation, allocation concealment, selective reporting, blinding of participants and personnel, blinding of outcome assessment, incomplete outcome data, and other bias.* During the assessment of each article, if information related to a specific item was not available, the item was rated as unclear. The overall number of high or unclear risk of bias allowed a final judgment for each paper evaluated. For dosage studies, quality assessment analysis was not possible due to the heterogeneity in study design and research questions.

Results were then sorted by themes of interest: “frequency studies” or “dosage studies”. In order to answer our search question, all the pertinent outcomes were listed in two categories, clinical or physiological outcomes.

## Results

### Descriptive numerical analysis

A total of 4854 articles were identified from the literature search. Following the removal of duplicates (*n* = 954), 3868 papers were excluded and 32 fulfilled the selection criteria. No article was retrieved from the additional data sources. Figure 1 presents the flowchart of studies selection and inclusion.

Out of the 32 studies included for this scoping review, 22 were experimental studies, seven were RCT, two were crossover studies, and one was a non-randomized control trial.

Six studies [13–18] focused on frequency outcomes and compared the effects of a specific number of SM treatment (from 1 to 18) delivered over a given period of time (from 3 to 8 weeks). All these studies were randomized control trials conducted on human participants with either cervicogenic headaches [16–18], chronic low back pain [14, 15] or lumbar spine stenosis [13], with the main outcomes being self-reported levels of pain and disability. Duration of treatment varied between 3, 6 or 8 weeks. Two studies reported post-manipulation outcomes only whereas four studies also included a 12, 20, 24 or 52-week follow-up measure.

On the other hand, 26 studies focused on dosage outcomes where biomechanical parameters derived from the SM force-time profile were used to set and compare dosages. Twelve studies involved human participants [10, 19–29] and 14 were animal-based studies [30–43]. No cadaver studies were included following the study selection process since they did not meet our inclusion criteria. Spinal manipulations were delivered in different ways including the use of: [1] research-developed mechanical apparatus (*n* = 18); [2] manual high-velocity low-amplitude SM (*n* = 5); [3] common clinical tools such as activator or impulse devices (n = 5, 4] manual mobilizations based on Maitland grades (*n* = 2, 5] flexion-distraction table (*n* = 2).

**Fig. 2:**
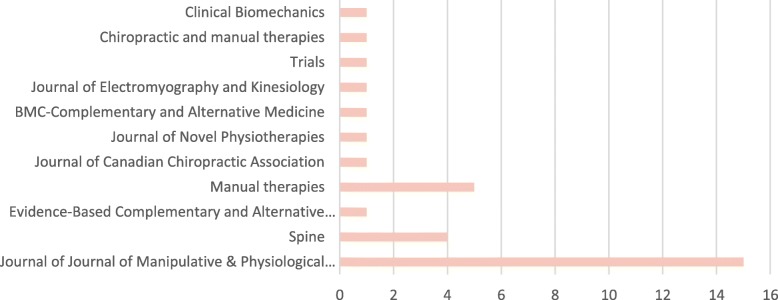
Number of included studies per peer-reviewed journal

The Journal of Manipulative and Physiological Therapeutics was the main scientific journal where the SM treatments frequency and dosage studies were published, with a total of 15 articles. Figure 2 presents all included studies based on peer-reviewed publication journals.

### Risk of bias assessment for randomized control trials

Seven randomized control trials were assessed for quality, of which six, reported on the effects of SM frequency [13–18] including four pilot studies [13, 14, 16, 18] and one reported on the dosage effects [21]. Four studies [13, 14, 16, 18] presented a high risk for other bias because they did not report an a priori sample size calculation and included between 24 to 80 participants. Five studies had one or two unclear risks of bias for reasons related to randomization, blinding of participants and personnel, or blinding of outcome assessment, which lowered the confidence in the overall reported effects. Finally, two studies were rated as having a low risk of bias [15, 17]. Table 1 summarizes the risk of bias for each of the RCTs.

**Table 1:**
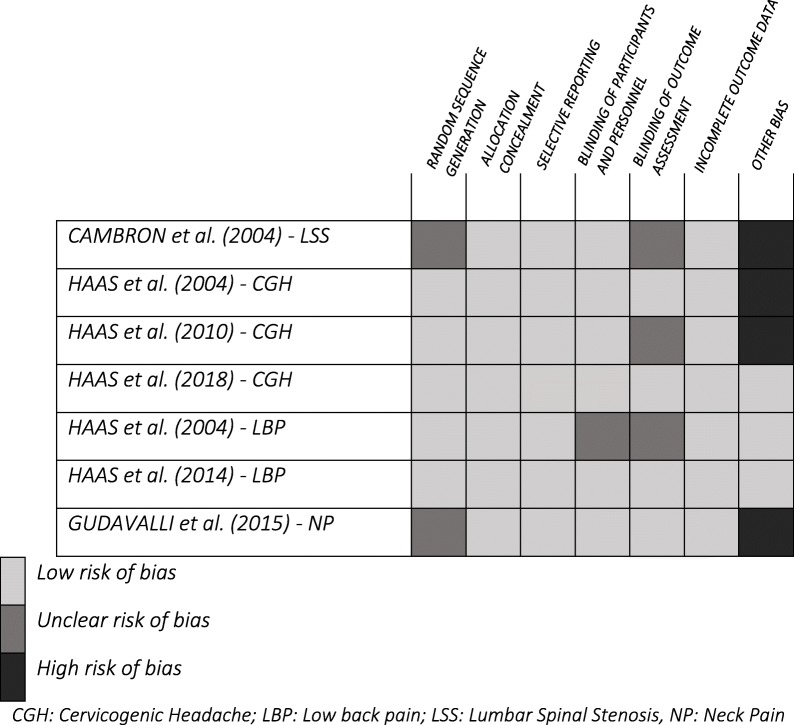
Cochrane Risk of bias tool assessment of randomized control trials

### Thematical analysis of findings

#### Frequency studies

##### Cervicogenic headache

Three studies investigated the effect of the number of SM on cervicogenic headache (CGH).

A randomized control trial by Haas et al. (2004) (*N* = 24) compared three different SM treatment frequencies (1, 3 or 4 times/week) over a 3-week period. Headache-related pain and disability decreased in people who received a total of 3 treatments and 4 treatments per week compared to one treatment per week. There was no significant between-group difference regarding neck pain and disability [16].

A randomized control trial from Haas et al. (2010) (*N* = 80) compared, over an 8-week period, four groups receiving either 8 or 16 SM treatments and 8 or 16 light massage (LM) treatments. Results showed that the two SM treatment frequencies yielded similar results for CGH pain and disability. Improvement in pain and disability were statistically and clinically higher in SM groups compared to LM. Again, the number of days with headache episodes decreased significantly more in SM groups compared to LM groups but no frequency effect was observed.

A pilot of a randomized control trial from Haas et al. (2018) (*N* = 256) compared the effect of different SM treatment frequency alone and combined with LM over a 6-week period [17]. The study involved 256 participants divided into four groups receiving: [1] 0 SM + 18 LM; [2] 6 SM + 12 LM; [3] 12 SM + 6 LM or [4] 18 SM + 0 LM. The results showed that the number of days with cervicogenic headaches decreased for all groups and that the most significant reduction occurred in 18 SM visits compared to LM. Differences between the three frequency groups, however, were not statistically different. There was no significant between-group difference regarding cervicogenic headache pain intensity and improvement was only observed for CGH disability of all 3 SM groups when compared to the control group.

##### Chronic low back pain

Two studies investigated the effect of treatment frequency in patients with chronic low back pain.

A pilot study from Haas et al., (2004) compared the number of SM treatments (alone or combined with physical modalities) over a 3-week period with a sample size of 70 participants. Four groups received 1 to 4 treatments per week. Low back pain intensity and associated disability were assessed over 12 weeks. Results showed that there was a significant number of treatment effect (regardless of the use of physical modalities) regarding disability. Indeed, compared to baseline, disability improved at 4 and 12 weeks with a higher effect of frequency (3 or 4 SM treatments per week) at 4 weeks compared to 12 weeks. Regarding pain intensity, higher treatment frequency led to a decrease in VAS score, at 12 weeks follow-up with results showing a significant interaction between the type and frequency of treatment. Results yielded important improvements in pain intensity when SM was associated with physical modalities and a higher number of treatment (3 or 4 SM treatments per week) [14].

In a randomized control trial including 400 participants, the same group investigated the dose-response relationship between the number of SM visits (LM compared to 1, 2 or 3 SM sessions per week) and clinical improvements in pain and disability over a 6-week period. They reported significant differences in outcomes favoring SM groups; however results showed no significant differences for pain intensity and disability between the various SM frequencies [15].

##### Lumbar spinal stenosis

One pilot study involving 60 participants focused on the effects of different flexion-distraction manipulation frequencies on pain and symptom severity in patients with lumbar spinal stenosis. Over a 6-week period, participants received 8, 12, or 18 treatments and were compared to a placebo group (low level laser therapy and simulated mechanically-assisted SM). Symptoms severity and disability were assessed up to 6 months. Compared to baseline scores, results showed that symptoms severity was significantly improved at completion of care and that the effect persisted at 3 and 6 months follow-up in the higher frequency groups (12–18 treatment over 6-week period. The study also reported that higher frequency of treatments (12–18 treatment over 6-week period) yielded an improvement in disability at 3 months follow-up. Similarly, the group that received 18 treatments showed significant improvements in disability at the end of care assessment and this difference remained 3 months later. Moreover there was no significant between groups differences for symptom severity, and no comparison were presented by the authors for disability. [13].

From all these studies, only one reported adverse event [17]. Identified adverse events were mostly classified as were all short term and classified as moderate resulting from treatment (SM or light massage). Adverse events related to SM were described as neck soreness, pain, stiffness, transient upper extremity pain/tingling, increased headache intensity, nausea or dizziness) and occurred in 40% of participants. The proportion of adverse events was similar for each treatment frequency (1, 2, or 3 SMT per week over a 6-week period).

#### Dosage studies

The majority of studies (25/26) [10, 19, 20, 22–31, 33–39, 41–44] reported on the physiological outcomes of SM and one reported on the clinical outcomes of SM in a neck pain population [21].

##### Clinical outcomes in humans

One pilot randomized control trial investigated the effects of three different manually-delivered cervical traction forces (low, medium and high) in 48 participants experiencing chronic neck pain. Results suggest that high-force tractions significantly improve neck pain compared to low-force tractions whereas, improvements in disability were significantly greater for medium and high-force tractions compared to low-force tractions [21].

##### Physiological outcomes in humans

The most commonly reported physiological outcomes were muscular response’s amplitude (*n* = 6), vertebral displacement (*n* = 5), and pain pressure threshold (*n* = 4). Table 2 summarizes the effects of SM based on outcome categories.

**Table 2:** Summary of the SM dose-physiological response relationship in human studies. (N=Newton)

Studies	Dosage parameters	Spine level	Sample size	Outcomes measures	Results
[19] *Colloca* et al*, 2003*	Thrust forces: *30 N, 150 N*	L4	N = 4	PAIN PRESSURE THRESHOLD	● NO DIFFERENCES
[23] *Krouwel* et al*, 2010*	Thrust forces: *50, 200 N*	L5	N = 30		● NO DIFFERENCES
	Thrust durations: *1,5 Hz or sustained pressure*				
[28] *Pentelka* et al*, 2012*	Thrust durations: *30s,60s*	L3	N = 19		● NO DIFFERENCES
[29] *Willett* et al*, 2010*	Thrust durations: *1 Hz, 2 Hz*	L1 to L3	N = 30		● NO DIFFERENCES
[19] *Colloca* et al*, 2003*	Thrust forces: *30 N, 150 N*	L1 to L3	N = 4	MUSCULAR RESPONSE AMPLITUDE	● Data suggest higher responses with maximum thrust force setting
[22] *Keller* et al*, 2000*	Thrust forces: *19,5 N,190 N*	Bilateral PSIS, sacrum, S1 and L5, L4, L2, T12 and T8	N = 40		● Increase after SM treatment
					● SMT showed a greater increasing than control group and sham treatment
[25] *Nougarou* et al*, 2014*	Preload forces: *5 N,50 N, 95 N, 140 N*	T6 to T8	N = 23		● Decrease during preload
					● Increase during thrust
[26] *Nougarou* et al*, 2016*	Combination of thrust forces / thrust durations: *57 ms/150 N, 80 ms/200 N, 102 ms,250 N, 125 ms/300 N*	T6 to T8	N = 25		● NO DIFFERENCES
[24] *Nougarou* et al*, 2013*	Thrust forces: *80,130,180, 255 N*	T6,T8	N = 26		● Increase in thrust phase and resolution phase
[10] *Pagé* et al*, 2016*	Thrust forces: *75 N,125 N,175 N, 225 N*	L3	N = 51		● Increase with increasing thrust force
[19] *Colloca* et al*, 2003*	Thrust forces: *30 N, 150 N*	L1 to L3	N = 4	VERTEBRAL DISPLACEMENT	● Data suggest an increase when greater force is applied
[20] *Colloca* et al*, 2004*	Thrust forces: *30 N, 88 N, 117 N, 150 N*	L3 to S2	N = 9		● Increase with increasing thrust force
[22] *Keller* et al*, 2000*	Thrust forces: 19,5 N,190 N	Bilateral PSIS, sacrum, S1 and L5, L4, L2, T12 and T8	N = 40		●increase after any treatment
					●SMT showed a greater increasing than control group and sham treatment
[25] *Nougarou* et al*, 2014*	Preload forces: *5 N,50 N, 95 N, 140 N*	T6 to T8	N = 23		●Linear decrease with force during thrust phase ● Increase in preload phase with increasing preload
[26] *Nougarou* et al*, 2016*	Combination of thrust forces / thrust durations: *57 ms/150 N, 80 ms/200 N, 102 ms,250 N, 125 ms/300 N*	T6 to T8	N = 25		● Increase in thrust phase with increasing thrust force
[27] *Pagé* et al*, 2014*	Thrust durations: *125 ms, 175 ms, 275 ms*	T7,T8	N = 20		● NO DIFFERENCES

##### Physiological outcomes in animals

The data extraction highlighted one major outcome described in 7 studies [30, 35–40]: muscle spindle activity (MSA). All studies showed that MSA increased with either decreasing thrust durations, increased applied forces or sometimes both [35–37]. Detailed results describing changes in muscle spindle activity and dosage effects are summarized in Table 3.

**Table 3:** Summary of the SM dose-physiological response relationship in animal studies

Studies	Dosage parameters	Sample size	Spine level	Muscle spindle activity - main results
[30] Cao et al., 2013	Thrust forces: 25, 55, 85% of BW	*n* = 112 cats	L6	● Consistent increase in MIF for 1 mm thrust amplitude.
	Thrust displacements: 1, 2 or 3 mm			●No specific trend associated to modulation in forces and displacements
	Thrust durations: 0,25,50,75,100,150,200, 250 ms			
[35] Pickar et al., 2006	Thrust forces: 33, 66, 100% of BW	*n* = 46 cats	L6	●Data suggest that decreasing thrust duration increases **Δ**MIF
	Thrust duration: 25, 50, 100, 200, 400 or 800 ms			● There is a threshold effect for duration for which the discharge greatly increases
[36] Pickar et al., 2007	Thrust displacement: 1 or 2 mm	*n* = 54 cats	L6	● Data suggest that decreasing thrust duration increases **Δ**MIF
	Thrust duration: 12.5, 25, 50, 100, 200, 400 ms			
				● Peak thrust amplitude (1 mm compared to 2 mm) influence **Δ**MIF
[37] Reed et al., 2013	Thrust forces: 25, 55, 85% of BW	*n* = 112 cats	L6	● Data suggest that decreasing thrust duration increases mean spindle discharge through range of forces.
	Thrust displacement: 1, 2 or 3 mm			● Through a range of force durations, increasing force seems to increase **Δ**MIF.
	Thrust durations: 25, 50, 75, 100, 200, 250 ms			● For most thrust duration, peak thrust displacement did not influence **Δ**MIF.
				● Increasing force rates increased MIF
[38] Reed et al., 2015	Thrust force: ranges from 68 N to 122 N	*n* = 1 cat	L7	● Data suggest that increasing force leads to increase **Δ**MIF.
[39] Reed et al., 2014	Preload variation: 18% or 43% of thrust force	*n* = 20 cats	L6	● Increasing longer preload duration (4 s compared to 1 s) increases **Δ**MIF.
	Thrust durations: 1 or 4 s (Thrust force: 55% of BW)			
				● A smaller magnitude of preload (18% compared to 43%) increases **Δ**MIF
				● The highest preload magnitude and longest duration led to a significantly greater mean decrease in resting spindle discharge
[40] Reed et al., 2017	Thrust force: 22 N, 44 N or 67 N	*n* = 6 cats	L6	● Data suggest that increase in force increased the time required until the first action potential.

*MIF* Mean Instantaneous Frequency, *ΔMIF* Changes in Mean Instantaneous Frequency, *BW* body of weight

Other physiological outcomes such as displacements, acceleration responses and muscle activation (EMGs) responses were studied using sheep [31, 33, 34]. Colloca et al. (2006) investigated the effects of varying posterior to anterior mechanical stimulation force-time profiles on lumbar spine. Variable durations or amplitudes of stimulation were applied. Descriptive results showed that EMG responses were higher for thrust duration of 100 ms and 200 ms compared to 10 ms. The displacement response and vertebral acceleration also increased linearly with force [31].

Two studies written by Keller et al., reported on acceleration responses outcomes [31, 33, 34]. The first one investigated SM impulses induced by an instrument. Fifteen sheep received three different force settings on the lumbar region. Results highlighted that when increasing force magnitude, acceleration responses increased in the 3 axes (axial, medio-lateral and posterior-anterior) [33]. The second one, compared three force settings (low, medium and high forces) of three types of mechanical instruments simulating SM. Stimulations were applied on the sheep’s T12 vertebrae. Results showed that acceleration responses and vertebral displacement increased in the 3 axes with increasing forces [34].

One study focused on the effect of SM therapy forces and durations on cat spine stiffness coefficient. Vaillant et al. (2012) divided cats into groups receiving a preload force or not, variable applied forces (ranges established according to body weight) and movement amplitudes (1, 2 or 3 mm). For each possible combination, eight different durations were applied on the lumbar region. Although results showed a complex significant interaction between duration, force and displacement amplitudes if SM therapy was preceded by a preload and under displacement control, consistent spinal stiffness modulation across thrust durations or thrust forces could not be identified [43].

Finally, in 2014, Reed et al. studied if variable SM thrust forces could alter mechanical trunk response thresholds in wide dynamic range and/or nociceptive specific lateral thalamic neurons [41]. This protocol was tested on rats’ lumbar spine. Three thrusts were randomly delivered with varying forces magnitudes (0, 55% or 85% body weight) for 100 ms. Electrophysiological activity of wide dynamic range and/or nociceptive specific lateral thalamic neurons was recorded. Results suggest that for nociceptive specific lateral thalamic neurons, dorsal-ventral thrust forces corresponding to 85% body weight increased mean trunk mechanical threshold compared to the control condition (0% body weight) but not compared to 55% body weight. There were no significant differences for wide dynamic range neurons.

## Discussion

This scoping review investigated the effect of SM therapy frequency and dosage on clinical and physiological outcomes. Our main objective was to identify and report all clinical and physiological outcomes specific to SM frequency and dosages effects. From all the included articles, we were able to establish that most studies focused on dosage effects (26 dosage studies compared to 6 frequency studies). Fourteen studies involved animals, while 18 studies were conducted on human participants. The most commonly addressed conditions in human studies were cervicogenic headache followed by low back pain. Study designs were heterogeneous and involved clinical and experimental studies. The effects of SM dosages were by far most commonly studied compared to treatment frequencies. To answer our main research question, outcomes were categorized into clinical or physiological outcomes. All human-based clinical trials investigating the effects of dosage (*n* = 1) or frequency (*n* = 6) included both pain and disability outcomes. On the other hand, physiological outcomes most commonly included muscle spindle activity, muscular response activity, vertebral displacement, pain pressure threshold and acceleration responses. Based on the studies included in this review, none of the human studies investigated the relationship between physiological outcomes and clinical outcomes.

### Frequency effects

In order to appreciate the clinical relevance of frequency effects, results should be interpreted considering the minimum clinically important difference (MCID) for pain and disability outcomes. Although three studies reported statistically significant frequency effects [15, 17, 45], only two studies described their results using clinically meaningful thresholds [15]. Indeed, only two RCT (Haas et al., 2014 [15] and 2018 [17]) reported clinically relevant effects, but these effects were present only when SM was compared to the control group (not or for frequency effects). These two studies showed that compared to no treatment or light massage, SM therapy had durable benefits for neuromusculoskeletal disorders-related pain and/or disability if the treatment is repeated over a period of time. When frequency effects are considered for patients with back pain or headache, trends in improvement were observed and indicated that increasing the number of SM visits in a short period of time (few weeks) decreased pain and improve disability as well as reducing the number of days with headaches episodes.

### Dosage effects

Four studies involving human participants showed that vertebral displacement and muscular amplitude responses increased when higher SM forces were applied whereas modulation of SM dosage did not seem to modify pain pressure thresholds. Despite consistent changes in physiological responses due to dosage effects, the association between these changes and clinical outcomes remains unknown. Only one paper investigated the effect of dosage on clinical outcomes and showed an improvement in pain and disability when medium or high force tractions were applied (compared to low force traction). However, the high risk of bias (Table 1) identified for this specific RCT refrains us from any definitive conclusion with regard to SM dosages and clinical outcomes.

Five studies focusing on muscle spindle discharge in animals consistently reported an increased mean frequency discharge when higher thrust forces were applied; and similar trends were seen for shorter thrust duration [30, 35–37, 39, 40, 46]. Muscle amplitude response assessed with EMGs was studied in one animal study and showed increased EMG response amplitudes when higher forces and longer thrust durations were applied [31]. Although animal models may have similar biomechanical properties with humans, anatomical factors (geometry and morphology) as well as loading characteristics of spinal structure are known to differ between such models and human spine [34]. A few studies attempted to reproduce a range of forces similar to clinically relevant SM in humans but the relative “clinical relevance” of the SM characteristics used in animal models was often reported as one of the challenges in data interpretation [39, 41]. Moreover, the use of different anaesthetics may have altered muscle function differently and again may have limited the generalization and interpretation of SM dosage effect studied in animal models. Animal studies provide valuable information when invasive procedures are needed and, for ethical reasons not possible in humans. Results, however, should be interpreted with caution, as they may not always reflect SM characteristics and effects in humans.

### Limitations

The first limitation that should be considered is the various operational definitions used for spinal manipulation dosage and frequency in the original studies. Some studies may have been missed or excluded due to the lack of consensus with regard to these definitions. A recent paper by Groeneweg et al. recommended a list of criteria to standardize the reporting of SM intervention [47]. Indeed, some studies did not use the definition described in our method for dosage and frequency terms in the same definition as described in our method. Although the authors proposed a clear definition for frequency, the definition for dosage remains ambiguous and seems to encompass time spent in therapy by the patient and efforts expended by the therapist during treatment sessions, two elements that seem to be related to overall care dosage rather than specific SM parameters [47]. Moreover, some of the clinical trials may have been underpowered as only two clinical studies adequately reported sample size determination. Lack of power in clinical studies may have led to inconsistent and sometimes misleading results and interpretations.

In addition, a comparison between studies could not be performed due to heterogeneity of SM uses across studies. In some studies, lack of SM standardization between conditions or groups within a given study may have been an issue [23, 29]. Finally, for 23 dosage studies, SM was delivered by a mechanical device simulating SM for which dosages were quantified. Although these devices were, in a few instances, designed to simulate clinician’s SM performance. Such device may not reflect the manual SM or mobilization most commonly performed by clinicians. According to Beliveau, only 23% (Interquartile range: 14.0–38.0) of chiropractor use instrumented assisted SM compared to 79% of chiropractors using manual SM [7].

### Research recommendations

Considering the high heterogeneity of the included studies regarding design, populations, conditions, outcomes and SM delivery (manual or mechanically assisted), it was not possible to determine optimal dosages and frequencies for the treatment of spinal conditions. As previously recommended future studies should provide detailed information with regards to SM, including treatment frequency and dosage. When possible, SM dosages should be described using treatment characteristics derived from the force-time profile. Several studies investigating SM motor control and learning have used force-sensing technologies to quantify SM biomechanical parameters [48, 49]. Such technologies should be considered in clinical trials evaluating not only dosage and frequency effect, but also in any study investigating clinical effects of manual therapy. The true dose-response relationship between SM biomechanical parameters and clinical outcomes could then be investigated.

## Conclusion

The results of this study showed that SM dosage and frequency effects have been mostly studied over the past two decades. Definitions for these two concepts are, however, heterogeneous across studies. Based on limited evidence, results suggest that treatment frequency does not significantly impact clinical outcomes during and following SM treatment period. However, additional work is likely to modify the current state of knowledge and a definitive conclusion at this time would be untimely. Dosage effects clearly influence short term physiological responses to SM treatment, but the relationship between these responses and clinical outcomes remains to be elucidated.

## Abbreviations

CGH: Cervicogenic Headache
DALYs: Disability-Adjusted Life Years
EMG: Electromyograms
MCID: Minimum Clinically Important Difference
RCT: Randomized Control Trial
SM: Spinal Manipulation
